# A novel, nurse-led ‘one stop’ clinic for patients with liver cirrhosis results in fewer liver-related unplanned readmissions and improved survival

**DOI:** 10.1186/s12876-023-02986-y

**Published:** 2023-10-16

**Authors:** Eric Kalo, Asma Baig, Emily Gregg, Jacob George, Scott Read, Wai-See Ma, Golo Ahlenstiel

**Affiliations:** 1 Blacktown Clinical School and Research Centre, School of Medicine, Western Sydney University, 18 Blacktown Road, Blacktown, NSW 2148 Australia; 2 Blacktown Hospital, Western Sydney Local Health District, Blacktown, NSW 2148 Australia; 3 Storr Liver Centre, The Westmead Institute for Medical Research, University of Sydney, Westmead, NSW 2145 Australia; 4 Westmead Hospital, Western Sydney Local Health District, Westmead, NSW 2145 Australia

**Keywords:** Cirrhosis, Nurse, Clinic, Readmissions, Mortality

## Abstract

**Objective:**

Delivering effective secondary preventive and integrated care has the potential to break the *revolving-door phenomenon* of frequent readmissions in patients with advanced chronic liver disease. To address this, we launched the *Care Coordination of Liver Disease* (CCoLD) pilot, a novel nurse-led cirrhosis clinic in Western Sydney.

**Methods and analysis:**

Following an index presentation to Blacktown or Mount Druitt hospitals (BMDH), patients (*n* = 89, matched by age, sex, and MELD-NA) were consecutively either followed up by the CCoLD clinical nurse consultant (intervention cohort) or received standard care (control cohort). Controlled evaluation of the impact of the nurse-led clinic was carried out for a 3-month period including readmission rates, survival, and cost effectiveness.

**Results:**

The inaugural nurse-led clinic led to improvement in patient-level outcomes including a reduction in unplanned liver-related readmissions (2.08% for intervention cohort vs 12.2% for control cohort, *p* < 0.01), and mortality at 30 days (0% for intervention cohort vs 7.3% for control cohort, *p* = 0.03). Similar trends were observed at 90 days from index discharge. No deaths were observed in the intervention cohort as compared to the control cohort at 90 days (0% versus 7.3%, *p* = 0.03), while unplanned liver-related readmissions were 10.41% for the intervention cohort vs 19.5% for the control cohort (*p* = 0.115). Moreover, time to readmission was significantly longer in the intervention cohort, resulting in an overall cost-effective intervention.

**Conclusion:**

These findings highlight the significant impact of optimised care-coordination. A nurse-led clinic can deliver patient-centred, goal-directed, and cost-effective secondary prevention and care. A multicentre randomised trial for wider evaluation of these findings is warranted.

**Supplementary Information:**

The online version contains supplementary material available at 10.1186/s12876-023-02986-y.

## Introduction

Chronic liver disease (CLD) represents a considerable health burden in Australia. CLD is particularly common in low-income areas of New South Wales (NSW, Australia) such as Blacktown and Mount Druitt. The common drivers are alcohol, chronic viral hepatitis B (HBV) and C (HCV) and metabolic dysfunction-associated steatotic liver disease (MASLD). It is associated with high morbidity and mortality resulting in significant high healthcare burden due to frequent early, unplanned hospital admissions [1–3]. Published data estimates that around 50% of hospitalised patients with CLD (whether due to variceal bleeding, ascites, hepatic encephalopathy, or alcohol misuse) will be readmitted within 90 days [4, 5]. Despite effective treatments, CLD related mortality and readmission rates in Blacktown-Mount Druitt area remains 70% higher than for NSW overall [6]. This *“revolving door”* phenomenon of frequent early rehospitalizations of patients with advanced chronic liver disease arises from the interaction of numerous, often multilayered, and challenging factors spanning from severity of CLD, its aetiology to failures in the healthcare system. This is due to unstructured patient follow-up, long waiting lists for specialist appointments, low health literacy and lack of knowledge by general practitioners of management of CLD in the community [7]. In addition, the chronic care model for these patients is fundamentally structured around hepatologists who are often time-constrained when aiming to deliver quality, holistic and personalised chronic care management.

A coordinated care approach for patient with cardiac failure for instance had been associated with cost savings, reduced burden on hospital system and reduced rates of morbidity. Likewise, coordinated clinical care models for other chronic disease management have been associated with a range of improved surrogate patient outcomes including reduced hospitalisation and readmissions due to improved symptom management and earlier escalation in the community [8]. A nurse care navigator has been shown to effectively improve triaging for outpatient clinics, linkage to primary care in the community and providing relational and informational continuity of care interventions necessary for retention of patients in disease management programs [9–11].

We therefore hypothesized that delivering effective secondary preventive and integrated care could break this revolving-door phenomenon of frequent readmissions [12–14]. For this, a coordinated clinical care protocol was proposed: The *Care Coordination of Liver Disease* (CCoLD) initiative is a pilot project of a nurse-led model of care at Blacktown Hospital provided to patients with diagnosed liver cirrhosis. The service directly engages with patients and their carers to coordinate and deliver patient-centred, early, evidence-based, and structured interprofessional collaborative clinical care with emphasis on self-care actions.

## Methods

### Intervention

**Fig. 1:**
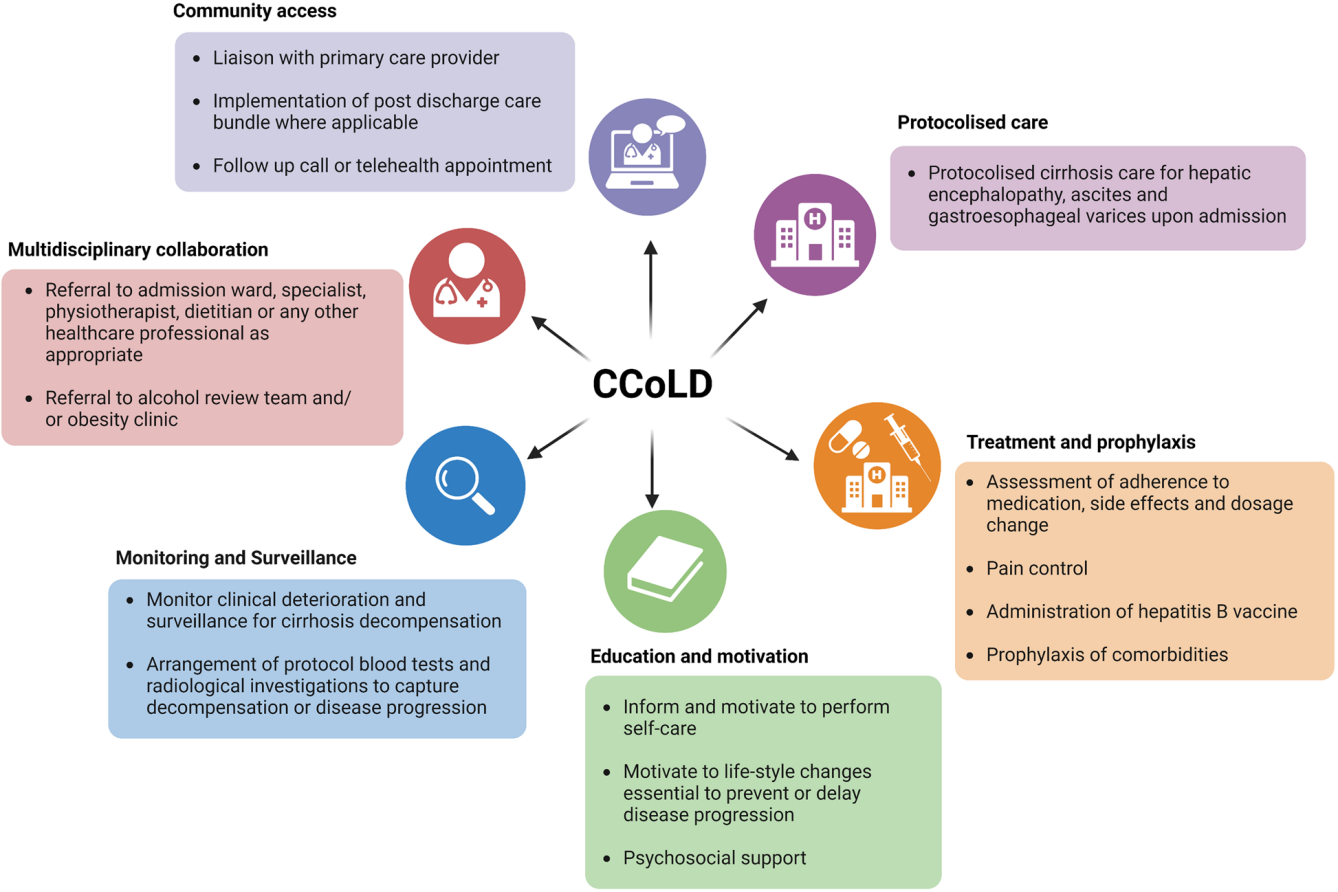
An overview of nurse-assisted interventions. The *Care Coordination of Liver Disease* (CCoLD) initiative is a pilot project of a nurse-led model of care at Blacktown Hospital in Western Sydney, Australia. The service directly engages with patients and their carers to coordinate and deliver patient-centred, early, evidence-based, and structured interprofessional collaborative clinical care with emphasis on self-care actions

Following an index hospital presentation to Blacktown or Mount Druitt Hospitals with liver cirrhosis, patients were consecutively either followed up in the nurse-led clinic (intervention cohort) or received standard hepatology specialist-led care as an inpatient or outpatient as required (control cohort). The intervention included goal-directed, protocolised cirrhosis care for hepatic encephalopathy, ascites, gastroesophageal varices, alcohol and substance misuse, malnutrition, psychosocial and post discharge care. Given the nature of the interventions, blinding participants and staff was not feasible. The nurse-led intervention compromised of one-stop visit to nurse-led cirrhosis clinic at Blacktown hospital or patients were visited by a nurse participating in the study during hospitalisation. The intervention lasted 30 min and was carried out during hospitalisation, and within 7 days post discharge when the patient visited the nurse-led clinic. An overview of the nurse-assisted interventions is provided in Fig. 1. The nurse involved in the study was proficient in the field of liver diseases, with 9-year-experience from hepatology inpatient or outpatient care and having a holistic understanding of liver cirrhosis. Demographic, vital signs and extensive laboratory data panel were recorded in all patients of the intervention cohort. Nurses played a key role in the initiation and maintenance of healthy lifestyle behaviours, coaching patients and their families in care planning and goal setting. The inclusion of nurses in the interprofessional teams facilitated the early involvement of allied healthcare professionals such dietitians (for restriction of sodium and/or water, specific dietetic measures for hepatic encephalopathy), physiotherapists, psychologists and social workers and further spurred the introduction of other health professionals and referrals as appropriate (alcohol review team and/or obesity clinic referrals). Moreover, the nurse worked with patients and caregivers to teach them how to recognise early symptoms, how to contact healthcare professionals. The patients within the intervention cohort were comprehensively assessed for potential complications or worsening of disease especially during post-discharge period or at discharge by activating discharge care bundles. The comprehensive assessment included observing the signs of decompensation (for instance, accumulation of fluid, gastroesophageal bleeding, alterations in mental status, jaundice), risk prevention and prophylaxis of comorbidities (frailty, malnutrition, sarcopenia and risk of falls assessments, COVID-19, pneumococcal pneumonia, influenza, and tetanus vaccinations, serologic testing for hepatitis A and B, screening for gastroesophageal varices), and other symptoms that may place additional stress on the patient. In addition to specific signs of the disease, patients were monitored for other symptoms, such as pain, breathlessness, difficulties sleeping, and/or anxiety. Further compliance to treatment and potential side effects were monitored (reviewing the dose of diuretics for the control of ascites and/or of lactulose for the treatment and/or prevention of HE, non-selective beta-blockers for the prevention of gastrointestinal bleeding, norfloxacin or trimethoprim-sulfamethoxazole for the prophylaxis of spontaneous bacterial peritonitis). In addition, nurses have facilitated real -life laboratory and necessary radiological investigations, performed hepatic elastography to capture disease progression. A hepatic ultrasound examination was performed for all patients due to increased risk of development of hepatocellular carcinoma. Moreover, psychosocial factors were explored to identify additional needs related to an increased dependency on assistance, fears of death, among others. Patients of the intervention cohort who were hospitalised were managed according to recent guidelines and recommendations. Patients of the intervention cohort-maintained linkage to care post hospitalisation via follow-up call or telehealth appointment or visit to the clinic during the first week or as needed during the study period. Primary care providers were notified of laboratory and procedural investigations in real-life, treatment plans or any changes to them.

Standard care (control cohort) included inpatient or outpatient visit or telephone follow-up by physicians, gastroscopies, ascites drainage, registered nurse telephone counselling by a nurse not participating in the study. In this cohort, after hospital discharge, the responsibility for the management in keeping with existing guidelines and protocols was entrusted to the primary physician with the on-demand support of specialist physicians.

### Study population

We included 89 individuals > 18 years-old with cirrhosis diagnosed using standard clinical criteria, radiological evidence consistent with cirrhosis, or transient wave elastography (> 20 kPa), and/ or liver biopsy. If a patient with chronic liver disease had endoscopic or radiological evidence of varices, or platelet count < 150,000/mm^3^ and aspartate aminotransferase/alanine aminotransferase ratio > 1, they were also included. For the purpose of this analysis, we included 41 control patients and 48 in the intervention cohort. The sample size was determined using the Cochran formula for sample size calculation. With an estimated prevalence of admissions for liver cirrhosis during three-month period and readmission rate up to 50%, a Z-score at 95% confidence level (1.96), and a level of precision of 0.05, the minimum sample size was calculated to be 80.

**Fig. 2:**
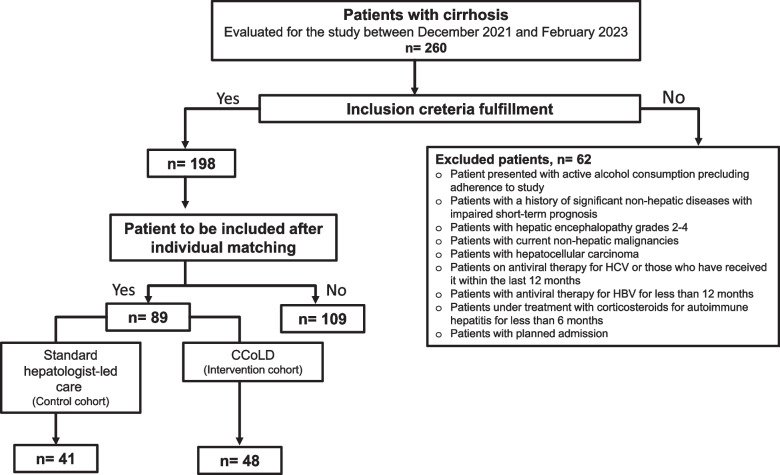
Flow diagram for study participants. Abbreviations: CCoLD, *Care Coordination of Liver Disease* initiative; HCV, Hepatitis C virus; HBV, Hepatitis B virus

Patients were excluded if they were: a) presented with active alcohol consumption expected to preclude correct adherence to study b) patients with a history of significant non-hepatic diseases with impaired short-term prognosis (heart failure NYHA Grade III/IV, COPD GOLD C or above, dementia, stroke with sequelae, severe psychiatric disease, and renal failure requiring dialysis) c) patients with hepatic encephalopathy grades 2–4 because of the need for sufficient time to exclude other conditions that resemble hepatic encephalopathy at admission and to correct for precipitating events. d) patients with current non-hepatic malignancies including solid tumours and hematologic disorders e) Patients with hepatocellular carcinoma, except for patients with early HCC (BCLC-0 or BCLC-A) or patients with previous history of HCC and absence of recurrence 2 years after treatment f) patients on antiviral therapy for HCV or those who have received it within the last 12 months as positive effect on clinical decompensations may be seen after long period of antiviral therapy g) Patients with antiviral therapy for HBV for less than 12 months to avoid the interference of the effect of the etiological treatment. h) patients under treatment with corticosteroids for autoimmune hepatitis for less than 6 months i) TIPS insertion within 6 months prior to study inclusion as TIPS placement can change the natural history of cirrhosis (improve survival and reduce decompensation episodes) k) acute-on-chronic liver failure j) planned admission (Fig. 2. Flow diagram for study participants).

A total of 260 patients with cirrhosis were evaluated for the study between December 2021 and February 2023.Only 198 patients fulfilled the inclusion criteria. Following an index presentation to Blacktown or Mount Druitt hospitals (BMDH), patients (matched by age, sex, and MELD-NA score) were consecutively either followed up by the CCoLD clinical nurse consultant (intervention cohort, *n* = 48) or received standard care (control cohort, *n* = 41). Patients of both cohorts were followed up to 90 days post hospital discharge.

### Ethics approval and informed consent

Ethics approval was obtained from the Human Research Ethics Committee at Western Sydney Local Health District (Ethics Approval 2021/ETH00149). All research was conducted in accordance with the Declarations of Helsinki. All participants signed informed consent before start of this project.

### Outcomes

Primary outcome measures included time to first readmission, rate of readmission and mortality (at 30 days and 90 days). Unplanned liver-related readmission was defined as any unscheduled readmission for ascites, hydrothorax, or renal failure related to liver disease, hepatic encephalopathy and oesophageal or gastric variceal bleeding or any combination thereof, requiring hospitalisation. Our analysis also included economic evaluation of CCoLD project including its cost effectiveness and financial sustainability of CCoLD as secondary outcome. Healthcare resource use, specifically in relation to liver cirrhosis, is derived through clinic audits and includes health professional consultations (primary and secondary care), hospital admissions (day care, inpatient stays, emergency department presentations), investigations, and treatments including over-the-counter medications. Informal home care, patient time when using health services and transportation costs were not accounted for.

### Statistical analysis

Clinical data including demographics, primary diagnosis at admission and stage of liver disease were analysed. Variables are presented as either mean with standard deviation or median with an interquartile range (IQR). Student’s t-test was used to compare continuous variables, and the chi squared test or Fisher’s exact test was used to compare categorical variables. All *P*-values were two-tailed, and *P*-values of less than 0.05 were considered statistically significant. IBM SPSS® Statistics (version 28.0.1.1; IBM Corp., Armonk, NY, USA) was used for statistical analyses. The potential confounding effect of co-morbidities was avoided by matching the patients for the chronic disease score based on diagnosis, namely the Charlson index, which was adjusted for patients with chronic liver disease.

## Results

We carried out an evaluation of the impact of the nurse led clinic for a 3-month period. There were no significant differences in age or sex at index presentation between cohorts. The main aetiology of liver cirrhosis was alcohol-related liver disease (ALD) followed by Metabolic dysfunction-associated steatotic liver disease (MASLD) in both cohorts. Mean model for End-stage Liver Disease – Sodium score (MELD-Na) at recruitment was 15.39 and 13.49 (*p* > 0.05) in control and intervention groups, respectively (Tables 1, S1).

**Table 1:** Characteristics of control and intervention cohorts

Characteristic		Intervention	Control	*p*-value*
		**(*n* = 48)**	**(*n* = 41)**	
**Gender**	*Female (%)*	12 (25%)	14 (34.14%)	ns
**Age**	Mean [Range]	62.8 [18–83]	62.4 [43–89]	ns
**MELD-Na**	Mean (SD)	13.5 (5.41)	15.4 (4.64)	ns
**Aetiology**	*ALD*	19	29	
	*HBV*	3	1	
	*HCV*	3	3	
	*MASLD*	15	5	
	*PBC*	2	1	
	*Cryptogenic*	5	0	
	*others*	1	2	
**CCI**	*Points*	4	4	ns

Data is presented as mean and percent (%)

*Abbreviations*: *ALD* Alcohol-related liver disease, *CCI* Charlson Comorbidity Index, *HBV* Hepatitis B virus, *HCV* Hepatitis C virus, *MASLD* Metabolic dysfunction-associated steatotic liver disease, *ns* not significant, *PBC* Primary biliary cirrhosis, *SD* Standard deviation

^*^p-value < 0.05 is considered statistically significant

**Fig. 3:**
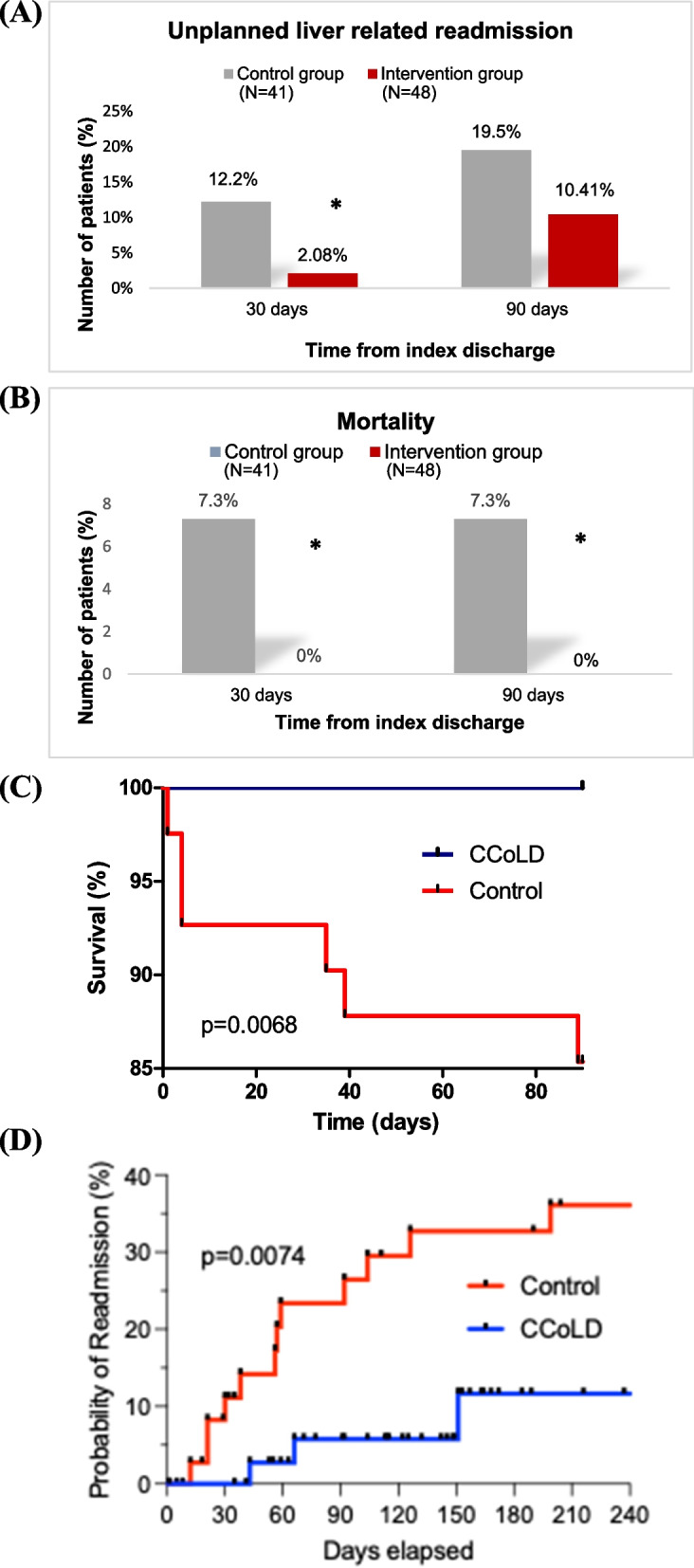
Outcomes of the CCoLD initiative. Nurse led clinic led to: **A** A significant reduction in unplanned liver-relate readmissions and **B** Favourable impact on patient survival. **C** Kaplan–meier survival curves for CCOLD and control cohorts (**D**) Probability of readmission in percent (%) for control cohort versus CCoLD over time, blue curve = intervention group, *n* = 48; red curve = control group, *n* = 41, (*p* = 0.0074). *p value < 0.05 is considered statistically significant

As illustrated in Fig. 3A, the intervention cohort experienced fewer readmissions at 30 days (2.08% vs 12.2%; *p* < 0.01), while at 90 days, a similar trend was observed (10.41% versus 19.5%, *p* = 0.115). Ascites and hepatic encephalopathy were the main causes of readmission in both cohorts at 30 and 90 days. Mortality for the control and intervention groups was 7.3% and 0% (*p* = 0.03) at 30 days, respectively. No deaths were observed in the intervention group as compared to the control group at 90 days (0% versus 7.3%, *p* = 0.03) (Fig. 3B). Patients of the intervention group showed better overall survival (Fig. 3C). Furthermore, a longer time required to emergency readmission was observed among those in the intervention cohort compared to the control cohort (*p* = 0.007) (Fig. 3D).

The nurse-led clinic was cost-effective with number needed to treat (NNT) being 10 to prevent 1 readmission within 30 days or four over the entire study period to prevent one readmission. The average cost of hospitalisation per patient with liver cirrhosis at Blacktown Hospital is at least AUD $22,000 and the nurse-led clinic prevented at least 9 readmissions. This represents a cost saving measure of AUD $198,000 over the 9 months of this study. Given that the clinical nurse consultant was funded at a cost of AUD $134,000 for 12 months, the project was cost effective. Costs attributable to global management of patients of the intervention cohort were significantly lower than the control cohort. All cost estimates associated with intervention and hospitalisation were validated by the Western Sydney Local Health District (WSLHD) finance department.

**Fig. 4:**
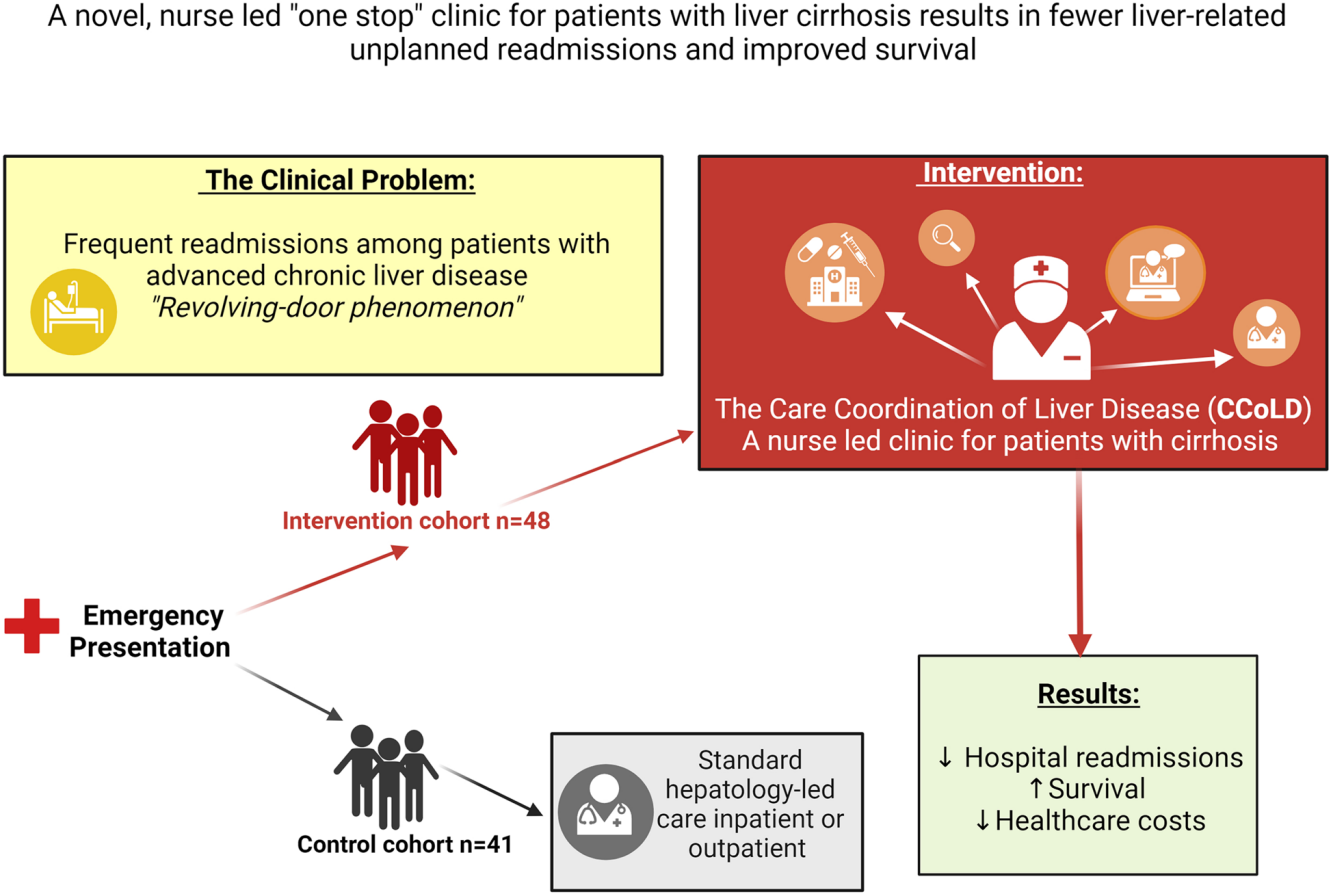
Schematic diagram showing key aspects of the CCoLD initiative (a nurse-led model of care) and its significant impact. Abbreviation: ED, Emergency department

Furthermore, the nurse-led clinic enabled patients to receive outpatient care in a non-admitted setting through a day medical unit. These patients would have required an admission had their condition deteriorated or not been diverted to outpatient services. Moreover, CCoLD improved attendance to specialist clinics and allowed earlier escalation of care related issues to medical staff prior to clinic appointments and facilitated prioritisation of hospital resources for complicated cases [15]. Figure 4. Summarises key aspects of CCoLD initiative and its significant impact.

## Discussions

Coordinated clinical care models have been largely successful in disease management of numerous chronic diseases such as diabetes mellitus, heart failure and chronic obstructive pulmonary disease [16]. Nevertheless, the role of nurses in the care of patients with chronic liver diseases remains widely underdeveloped in many countries [17, 18]. This is unfortunate, as nurse involvement in CLD management has been shown to limit liver-related emergency admissions by improving symptom management, triage for outpatient clinics and earlier escalation in the community [8, 11, 19]. The novelty of CCoLD is its integration of two principles of chronic disease management, namely implementing a modern delivery chain with community access and multidisciplinary care [20]. Nurse-led clinics represent an innovation in health service provision and ultimately can improve care and prevent fragmentation of care for this difficult to treat cohort of patients [21]. In Western Sydney there is an established example of a similar service, namely the Diabetes Rapid Access and Stabilisation Services (RAAS). This innovative model facilitates state-of-the-art care for patients with chronic disease and has proven successful in replacing existing traditional hospital-based care amidst COVID-19 pandemic.

In the context of a growing burden of advanced chronic liver disease and a pressure to reduce hospital readmissions, our findings provide compelling evidence that effective early interventions such as the introduction of a protocolised nurse service during hospitalisation of patients with cirrhosis or post discharge has significant overall benefits to patients. Furthermore, highlights the significance of care coordination models in achieving surrogate patient outcomes as proxies for continuity of care. Patients with cirrhosis without an early follow-up, a central element of transitional care- are at a particularly high risk for short-term mortality [22]. Moreover, early liver-related readmission is associated with higher 1-year mortality. A recent study has demonstrated that MELD-Na score and low haemoglobin are independent risk factors for early readmissions after index hospitalisation because of acute decompensation event [23]. These independent predictors of early readmission can be easily missed without programmes of closer surveillance post discharge such as nurse-led clinics. In addition to improved access, nurse-led clinic can provide at least equal or at times better outcomes compared to standard care with high levels of patient satisfaction and convenience [24, 25]. The improved accessibility offered by a cirrhosis nurse-led clinic has improved patient adherence to clinic attendance, translating into improved compliance with clinic appointments, endoscopic and radiological surveillance, earlier escalation of complications, and overall better disease management. Patients in nurse-led clinic are also more inclined towards accepting advice on alcohol substance misuse [26]. In addition, the education of patients and their caregivers, and telemedicine have shown encouraging results to reduce readmissions [27–29].

Readmissions rates can be amplified by low socio-economic factors [30]. However, this factor was minimal in our study. As a key enabler to access the hospital services for both cohorts is the availability of universal health insurance scheme. Moreover, the majority of recruited patients in our study were from Western Sydney area to minimise barriers to nurse-led clinic access such as transportation availability and location.

Limitations of our study include the small cohort sizes, and the possibility that unmeasured factors could have impacted the outcomes. For instance, our analysis did not rule out the independent impact of cirrhosis aetiology given that 71% of patients in the control cohort had alcohol-related liver disease compared to 40% in the intervention cohort. Moreover, our study employed a clinical nurse consultant with extensive multidisciplinary experience, and hence our findings might therefore be impacted by the level of nursing experience. Moreover, our stringent exclusion criteria of patients with characteristics that place them at high-risk for readmission (active alcohol consumption precluding adherence to study, creation of a transjugular intrahepatic portosystemic shunt within 6 months, and recent antiviral therapy, etc.) is another major limitation. All, these limitations could have led us to overestimate the benefit of CCoLD. Nonetheless, our findings highlight the significance of an adjunctive nurse-led clinics to standard physicians in liver cirrhosis in delivering goal-directed, cost-effective secondary prevention complementing the complex cirrhosis curative care. In conclusion, CCoLD has the advantage over standard care and the potential to address the unmet health demands from an ever-growing prevalence of liver cirrhosis as it enables clinicians, patients, and their caregivers to work collaboratively to identify treatments and other healthcare that best align with patients’ priorities. A large multicentre randomised controlled trial for wider evaluation of CCoLD long term impacts is warranted.

### Supplementary Information

**Additional file 1: Table S1.** Baseline characteristics of intervention cohort (*n*=48).

## Data Availability

The datasets used and/or analysed during the current study available from the corresponding author on reasonable request.
