# Supplementary material for: A novel, nurse-led ‘one stop’ clinic for patients with liver cirrhosis results in fewer liver-related unplanned readmissions and improved survival
Source: BMC Gastroenterol. 2023 Oct 16;23:356. doi: 10.1186/s12876-023-02986-y (PMC10578021; doi:10.1186/s12876-023-02986-y)
Supplement: Supplementary file 1 — Additional file 1: Table S1. Baseline characteristics of intervention cohort (n=48). [file 12876_2023_2986_MOESM1_ESM.docx]

**SUPPLMENTARY MATERIAL**

**Table S1. Baseline characteristics of intervention cohort (n=48)**

| **Characteristic** | | **Value** |
| --- | --- | --- |
| **Gender** | Female (%) | 14 (34.1%) |
| **Age** | Mean [Range] | 62.4 [18-83] |
| **MELD-Na** | Mean (SD) | 13.5 (5.41) |
| **Child-Pugh Class** | *A* | 26 (28.6%) |
|  | *B* | 16 (17.6%) |
|  | *C* | 5 (5.5%) |
| **ATSI** | *Aboriginal but not Torres Strait Islander origin* | 4 (8.3%) |
| **COB** | *Born overseas* | 27 (56.3%) |

Abbreviations: ATSI, Aboriginal and Torres Strait Islander peoples; COB, country of birth; CPS, Child Pugh score; MELD-Na, model for end-stage liver disease-Sodium; SD, standard deviation.
